# Build angle effect on 3D-printed dental crowns marginal fit using digital light-processing and stereo-lithography technology: an in vitro study

**DOI:** 10.1186/s12903-024-03851-4

**Published:** 2024-01-11

**Authors:** Engy Farag, Ahmed Sabet, Kamal Ebeid, Omar El Sergany

**Affiliations:** 1 Department of Fixed Prosthodontics, Faculty of Dentistry, The British University in Egypt, Cairo, Egypt; 2 Department of Fixed Prosthodontics, Faculty of Dentistry, Ain Shams University, Organization of African Unity Street, Cairo, Egypt

**Keywords:** Build orientation, Dental marginal adaptation, Digital light projection, Stereolithography

## Abstract

**Background:**

The effect of 3D printing technology and build angle on the marginal fit of printed crowns is unclear. The objective of this research was to use digital light processing (DLP) and stereo-lithography (SLA)-based 3D printing to construct single restorations with varied build angles and to analyze the crowns′ marginal fit.

**Methods:**

A prepared resin first molar was scanned utilizing an optical scanner. Three build orientations were used to construct the specimens: 0, 45, and 90º. DLP and SLA technology were used to produce the casting patterns. A digital microscope was used to measure the marginal gaps. The effect of build orientation was statistically analyzed by using Two-way ANOVA followed by pair-wise Tukey test.

**Results:**

Two-way ANOVA revealed a significant effect of printer technology and build angle on the marginal discrepancy of 3D printed crowns (*p* < 0.001). One-way ANOVA revealed that SLA printers (55.6 [± 13.59]) showed significantly better mean [± SD] marginal discrepancy in µm than DLP printers (72 [± 13.67]) (*p* < 0.001). Regarding build angle, one-way ANOVA revealed significant differences between the different angles. Tukeys post-hoc test revealed that 0° (48.5 [± 9.04]) had the significantly smallest marginal discrepancy followed by 45° (62.5 [± 8.05]) then 90° (80.5 [± 8.99]) (*p* < 0.001).

**Conclusion:**

The build orientation affects the marginal discrepancy of single crowns manufactured utilizing DLP and SLA.

## Background

The usage of digital technology nowadays in the field of restorative dentistry has increased significantly [1, 2]. Computer-aided design/computer-aided manufacturing (CAD/CAM) techniques are used by digital fabrication technologies in either subtractive (milling) or additive (3D printing) manufacturing (AM) [3]. AM has a distinct benefit over traditional milling techniques processes in that it generates virtually little waste, there are no limitations on the products geometric design, and accuracy of milled components is no more a concern [4–6]. This enables AM technologies to have a significant role in the mass manufacture of items with unique geometrical specifications [4].

The AM method differs based on the material used, with the digital light processing (DLP) approach including the building of a liquid photopolymer by UV light to achieve the end shape [7]. A very complicated structure is produced layer by layer directly from 3D data in a DLP build process. Depending on the final shape of the desired outcome, successive layers of liquid photoactivated monomer are subjected to UV light and cured. The DLP process creates a mask image dynamically and displays it on the resin surface using a digital micromirror device (DMD) [8, 9]. DMDs control the direction of light reflection by utilizing hundreds of thousands of freely moveable micromirrors. Each pixel in the picture corresponds to a single micromirror, the orientation of which may be changed depending on the geometry of the printed item [10].

The stereolithography (SLA) is often regarded as the most significant and widely utilized 3D printing technique in the world. Each layer in SLA is done by small lines created by a highly concentrated UV laser beam [11]. The polymer hardens as the laser traces the layer, leaving the surplus portions as liquid. When a layer is finished, a levelling blade is used to smooth the surface before applying the following layer. The platform is lowered by the thickness of the layer (usually 50–75 μm), and the next layer is created on top of the previously finished layers. This tracing and smoothing operation is repeated until the construction is finished. When the part is finished, it is lifted over the vat and drained. When the polymerization of one layer is complete, the build platform or resin tank slides up or down with relation to the layer’s thickness. The travelling direction is determined by whether the construction process is top-down or bottom-up. After the final cure, the surfaces are polished, sanded, and completed [12].

Because of the basic layer-by-layer production principle of 3D printing, build orientation impacts items in 3D printing in a fundamentally distinct manner than subtractive manufacturing [13]. Hong et al. stated that selecting the correct construction orientation improves volumetric accuracy, decreases manufacturing time and cost, and reduces the number of supports required for printing [14]. By making full use of the light source, one of the features that may improve the geometrical precision and structural quality of the final 3D-printed item is customizing build angle/orientation during the build process [15].

The cement space thickness and placement, for example, may be simply adjusted in the adaption of CAD models, and some researchers have employed a 30 μm cement space [16, 17]. However, a thicker cement spacing may be appropriate for therapeutic applications, like utilizing a luting substance with a high bonding strength, it might enhance crown and fixed dental prosthesis adaption [11, 18].

Several studies concluded that 3D printed crowns offer a better fit and higher precision than milled crowns [5, 19, 20]. Other studies also concluded that build angle have a direct effect on the accuracy of provisional crowns [6, 11]. Thus, the aim of this study was to evaluate the effect of 3D printing technology and build orientation on marginal accuracy of permanent crowns.

The first null hypothesis is that the marginal accuracy of crowns is not affected by the 3D printing technology. The second null hypothesis is that build orientation will not influence the marginal accuracy.

## Methods

In this in vitro study 60 full anatomical mandibular molar crowns were fabricated. Crowns were divided into two groups (*n* = 30) according to the printing technology (Group 1; DLP and group 2; SLA). Each group was divided into 3 subgroups (*n* = 10) according to the printing build angle (Subgroup A; 0°, subgroup B; 45°, and subgroup C; 90°). Crowns were later evaluated for marginal accuracy on their corresponding abutment tooth.

A mandibular first molar resin tooth (Nissin Dental, Kyoto, Japan) was reduced by 2 mm preparation on the occlusal surface and a total of 6° convergence angle with a 1 mm circumferential deep chamfer finish line. A model scanner (E2: 3Shape Copenhagen, Denmark) was used to scan the prepared molar. Once the scan data has been converted to STL format, CAD software (Galway 3.0; Exocad, Darmstadt, Germany) was used to design a single molar crown. Cement gap was set to 30 μm.

**Fig. 1:**
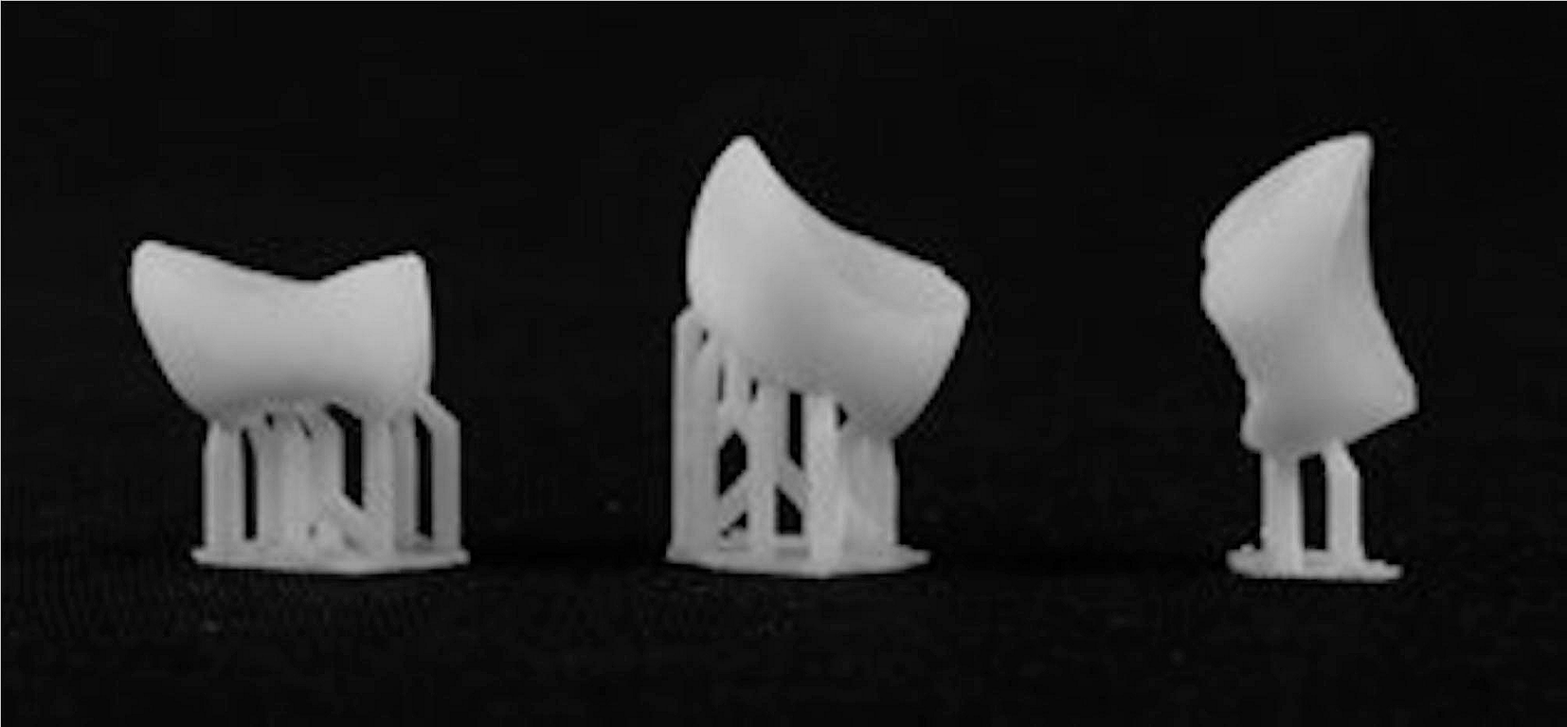
3D printed crowns with different build angles

The designed crown was imported into a software for 3D printing. For DLP crowns, 3D sprint software (3D Systems, Soesterberg, Netherlands) was used while the Preform software (Formlabs, Somerville, MA, USA) was used for SLA crowns. In the 3D printer software, the crowns were positioned on a platform and rotated in accordance with each construction direction. The 0° direction was when the crown’s occlusal surface set parallel to the platform. The crown image was rotated 45° along the long axis of the crown to get the 45° construction orientations. To support the vat photopolymerized cast pattern, cylindrical rods were affixed to the buccal cusps, lingual cusps, and marginal ridges. The crown lingual surface was placed perpendicular to the support framework for a construction angle of 90° (Fig. 1).

A DLP (5100; NexDent, Soesterberg, Netherlands) and liquid photopolymer (C&B; NexDent, 3D Systems, Soesterberg, Netherlands) were used to print 30 crowns. While the other 30 crowns were printed using stereolithography (Form 2; Formlabs, Somerville, MA, USA) in a hybrid composite resin material (Permanent crown resin; Formlabs, Somerville, MA, USA). The layer thickness for all samples was set to 50 μm.

Following the manufacturer’s instructions, the DLP prints were cleaned for 5 min with 96% 2-propanol (Emsure; Merck, Darmstadt, Germany), and then cured for 45 min using a light curing device (3D systems; LC-3D Print Box, Soesterberg, Netherlands).

In accordance with the manufacturer’s instructions, the stereolithography printed specimens were cleaned in 95% ethanol for 1 min and then post-cured for 30 min with a UV curing unit (S2; DWS, Thiene, Italy). After that, all the samples were preserved for ten days in a dry, light-proof box (Multiroir, Perigny, Fance) before being inspected.

Marginal discrepancy was assessed by measuring the vertical distance between each fabricated crown margin and corresponding abutment preparation finish line parallel to the tooth axis. Measurements were done for each crown along the circumference at the mid of buccal, mesial, lingual and distal surfaces. All the samples were examined at 40× magnification under the lens of USB digital microscope with a built-in camera (Scope Capture Digital Microscope, Guangdong, China) connected with an IBM compatible personal computer. A digital image analysis system (Image J 1.43U; National Institute of Health, Maryland, USA) was used to measure and qualitatively evaluate the gap width. All the imaging was captured, and measurements were done at the four sites. The mean of the four sites together (buccal, mesial, lingual, and distal) were recorded and taken as the final mean for each sample.

The data collected was checked for normal distribution using Kolomgrov-Smirnov and Shapiro–Wilk tests and analyzed using one-way and two-way analysis of variances (ANOVA), followed by Tukey’s HSD test (SPSS Statistics for Windows, Version 20.0.; IBM, Armonk, NY, USA) at a significance level of *p* ≤ 0.05.

## Results

Mean (SD) for all subgroups in µm are listed in Table 1. Two-way ANOVA revealed a significant effect of printer technology and build angle on the marginal discrepancy of 3D printed crowns (*p* < 0.001). One-way ANOVA revealed that SLA printers (55.6 [± 13.59]) showed significantly better mean [± SD] marginal discrepancy in µm than DLP printers (72 [± 13.67]) (*p* < 0.001). Regarding build angle, one-way ANOVA revealed significant differences between the different angles. Tukeys post-hoc test revealed that 0° (48.5 [± 9.04]) had the significantly smallest marginal discrepancy followed by 45° (62.5 [± 8.05]) then 90° (80.5 [± 8.99]) (*p* < 0.001).

**Table 1:** Mean (SD) and p- value of ANOVA for the marginal gap in microns of each printer with different angles

Build angle Printer type	0	45	90	P-Value
SLA	40 (1.63) a	55 (1.49) b	72 (1.2) c	< 0.00001
DLP	57 (1.24) a	70 (1.89) b	89 (2.62) c	< 0.00001

## Discussion

This study was conducted to evaluate the effect of 3D printing technology and build orientation on marginal accuracy of lower first molar crowns. As a result, the marginal fit varied depending on the construction directions. The null hypothesis was rejected based on the findings of the comparative examination of margin fitness for the two measurement methodologies.

Three different build angles were utilized to 3D print full-coverage dental crowns. Several steps were made to reduce and eradicate all possible handling and processing faults. In the center of the build platform, each crown was printed individually. The fabrication and post-curing processes were carried out by a single trained practitioner of the two technologies. Based on earlier research, the cement space was planned to be 30 μm [16, 17]. Thick cement space would have removed minor discrepancies between the modifications [11, 18, 21].

In terms of printing accuracy, quality, speed, affordability, and printer compactness, SLA and DLP technologies are the most often utilized 3D printing technologies in dentistry. As a result, they were the technologies of choice employed in this study. Meanwhile, the stereolithography technique is distinguished by its great manufacturing precision, which results in a more comfortable and exact fit of the dental crown. Curves, holes, and more complicated shapes are duplicated more easily and correctly, and the piece is manufactured exactly as specified, with no waste [22]. The minimum layer thickness was chosen for each 3D printer which was 50 μm.

The marginal discrepancy was measured using the digital microscope. This method is widely used, since it is nondestructive, there is no need for intermediate materials such as impression materials or luting cement in between and the measurements can be carried out at various sites [23, 24].

It is believed that build orientation affects the characteristics of photopolymerized items. Due to past research that produced FDPs in the forms of a bar or a crown using stereolithography or DLP, the 0º, 45º, and 90º build orientations were chosen [18, 21, 25, 26]. Park et al. reported that the 45º orientation of interim polymer-based FDPs’ marginal gap was clinically acceptable [27].

All specimens’ marginal gap, with different build orientation, was under the clinically permitted limits of 120 μm [28]. Specimens with different construction orientations may have marginal gap differences due to excessive polymerization at the intaglio buccal wall with different light penetration area [29–31]. The specimens’ marginal gaps of 90º were substantially larger than those of 0º and 45º specimens. Excessive polymerization is highest at the buccal intaglio surface of 90º samples because the buccal wall of the 90º sample is created by light entering a broader area with a shorter penetrating distance than that of the 0º and 45º samples [21].

Virtually cutting a 3D item into layers, which are then printed individually and stacked to copolymerize and gradually construct the desired object, is the first step in the 3D printing process. The “staircase effect” is a phenomenon that can happen when printing objects with inclined construction orientations. In this phenomenon, layers are printed gradually and the step edges between them result in errors [15, 32]. Therefore, it is reasonable to assume that the 45° and 90° orientations will produce less accuracy than the 0° orientation [33].

In specimens with different construction orientations, gravity may distort the area that hangs over the buccal and lingual walls, changing the size of the marginal gaps. These findings are in line with those of Ryu et al. [11], that a 180º building orientation is best (converted to 0º in the present study) but disagree with of Park et al. [27] who suggested a construction 45º orientation rather than 0º.

Other investigations also discovered the impact of the supporting structures. Yu et al. reported that a resin prosthesis produced using a SLA 3D printer had poor quality of the margins close to the support attachment and frequently had roughened edges [6]. The support attachment position varies with the construction angle. Unsupported sections might cause errors. If the support is connected near to the crown margin, it may cause harm during removal [18]. Alharbi et al. investigated the dimensional accuracy of the build orientation of dental crowns fabricated with a DLP 3D printer, the root mean square error (RMSE) was higher in the area with the support structure [18]. The support attachment position varies with the construction angle. Unsupported sections might cause errors. If the support is connected near to the crown margin, it may cause harm during removal. Meanwhile, the form of the layer created by the 3D printer differs according to the build angle which is a reason for the differences in the marginal fit. A DLP-based 3D printer polymerizes one layer at a time, any change in the layer form entails changes in the form and degree of polymerization shrinkage. SLA technology employs an ultraviolet (UV) laser to cure material point by point [25, 26].

The choice of construction angle is crucial since it influences the amount of support structures required, which may have an impact on the precision of the created components [18]. The number of surfaces that are self-supported increases and the support structures number decreases when the crown is rotated from a 90º construction angle to a 0º build angle. In the 90º construction angle, the support structure was placed close to the marginal area of crown. This enhances the possibility that when the support structures are removed, this important part of the restoration may undergo damage. Support was set back from the crown edges in the 45º construction angle. Since most of the printed crown’s surfaces were self-supporting, finishing and polishing required less time and support surface area.

In this research, the marginal gap mean values for the 0º and 45º build angles with the SLA, respectively, were 40 and 55 μm, while the mean value for the 90º build angle was 72 μm. The explanation of that is, as compared to the 90º angle, the crown’s angulation at 0º and 45º supplied the most self-supporting geometry.

Recent research that tested the geometric precision of the SLA-fabricated printed crown showed a maximum value of 0.042 mm, which is still smaller than the smallest variation observed with DLP [18]. This is due to the distinction between the two production procedures. The optical parameters of the system, such as the DMD device, lens quality, pixel size, and platform resolution, have an additional influence on the DLP printing precision [7, 9]. Additionally, these results support the claims made in a number of studies in the literature that the SLA method is one of the most precise manufacturing additive technologies. However, studies showed that there is no one 3D printing method that is better than another and that a well picked technology will perform the needed purpose [34, 35].

Although the results of this study maybe promising, there needs to be more investigations to determine the effects of numerous aspects, including platform position, support type, and layer thickness, that should be taken into account while printing crowns. Also, this study was done on single crowns, thus longer spans or intracoronal restorations may be further examined in future studies.

## Conclusions

1. Printing technology has a direct effect on the marginal accuracy of 3D printed permanent crowns.
2. Printing build angle has an effect on the marginal accuracy.
3. All tested variables showed results within the clinically acceptable range.

## Data Availability

The datasets used and/or analysed during the current study available from the corresponding author on reasonable request.
